# Machine Learning for Prediction of Adverse Cardiovascular Events in Adults With Repaired Tetralogy of Fallot Using Clinical and Cardiovascular Magnetic Resonance Imaging Variables

**DOI:** 10.1161/CIRCIMAGING.122.015205

**Published:** 2023-06-20

**Authors:** Ayako Ishikita, Chris McIntosh, Kate Hanneman, Myunghyun M. Lee, Tiffany Liang, Gauri R. Karur, S. Lucy Roche, Edward Hickey, Tal Geva, David J. Barron, Rachel M. Wald

**Affiliations:** Division of Cardiology, Peter Munk Cardiac Centre, University Health Network, University of Toronto, ON, Canada (A.I., C.M., T.L., S.L.R., R.M.W.).; Department of Medical Biophysics (C.M.), University of Toronto, ON, Canada.; Department of Medical Imaging, University Health Network (C.M., K.H., G.R.K., R.M.W.), University of Toronto, ON, Canada.; Department of Cardiovascular Surgery, Hospital for Sick Children (M.M.L., D.J.B.), University of Toronto, ON, Canada.; Department of Surgery, Division of Congenital Heart Surgery, Texas Children’s Hospital, Houston (E.H.).; Department of Cardiology, Children’s Hospital Boston and Department of Pediatrics, Harvard Medical School, Boston, MA (T.G.).; The Heart Institute, Hadassah Medical Center, Hebrew University, Jerusalem, Israel (R.M.W.).

**Keywords:** heart defects, congenital, machine learning, magnetic resonance imaging, prognosis, tetralogy of Fallot

## Abstract

Existing models for prediction of major adverse cardiovascular events (MACE) after repair of tetralogy of Fallot have been limited by modest predictive capacity and limited applicability to routine clinical practice. We hypothesized that an artificial intelligence model using an array of parameters would enhance 5-year MACE prediction in adults with repaired tetralogy of Fallot.

**Methods:**

A machine learning algorithm was applied to 2 nonoverlapping, institutional databases of adults with repaired tetralogy of Fallot: (1) for model development, a prospectively constructed clinical and cardiovascular magnetic resonance registry; (2) for model validation, a retrospective database comprised of variables extracted from the electronic health record. The MACE composite outcome included mortality, resuscitated sudden death, sustained ventricular tachycardia and heart failure. Analysis was restricted to individuals with MACE or followed ≥5 years. A random forest model was trained using machine learning (n=57 variables). Repeated random sub-sampling validation was sequentially applied to the development dataset followed by application to the validation dataset.

**Results:**

We identified 804 individuals (n=312 for development and n=492 for validation). Model prediction (area under the curve [95% CI]) for MACE in the validation dataset was strong (0.82 [0.74–0.89]) with superior performance to a conventional Cox multivariable model (0.63 [0.51–0.75]; *P*=0.003). Model performance did not change significantly with input restricted to the 10 strongest features (decreasing order of strength: right ventricular end-systolic volume indexed, right ventricular ejection fraction, age at cardiovascular magnetic resonance imaging, age at repair, absolute ventilatory anaerobic threshold, right ventricular end-diastolic volume indexed, ventilatory anaerobic threshold % predicted, peak aerobic capacity, left ventricular ejection fraction, and pulmonary regurgitation fraction; 0.81 [0.72–0.89]; *P*=0.232). Removing exercise parameters resulted in inferior model performance (0.75 [0.65–0.84]; *P*=0.002).

**Conclusions:**

In this single-center study, a machine learning-based prediction model comprised of readily available clinical and cardiovascular magnetic resonance imaging variables performed well in an independent validation cohort. Further study will determine the value of this model for risk stratification in adults with repared tetralogy of Fallot.

**Clinical Perspective**

The application of routine parameters obtained from clinical practice for accurate and efficient prediction of major adverse cardiovascular events (MACE) in adults after repair of tetralogy of Fallot is highly desirable. We present a novel machine learning model for prediction of MACE in adults with repair of tetralogy of Fallot using 2 preexisting institutional databases, which we named the artificial intelligence model derived from Toronto patient data (AiTOR). For AiTOR model development, readily available clinical and imaging variables were selected (unlike previously published risk scores based on invasive parameters or specialized measures). The AiTOR model demonstrated strong predictive capacity for 5-year MACE based on 57 variables extracted directly from the electronic health record (area under the curve, 0.82 [95% CI, 0.74–0.89]). Performance of the AiTOR model was superior to MACE prediction by conventional Cox (linear regression) modelling. Notwithstanding the real-world nature of the AiTOR model, predictive capacity for MACE matched or exceeded previously published risk scores. When the AiTOR model was restricted to the 10 strongest features (5 cardiovascular magnetic imaging variables [measuring right ventricular volumes at end-systole and end-diastole, biventricular ejection fraction, and pulmonary regurgitation], 3 cardiopulmonary exercise variables [assessing functional capacity], and 2 demographic variables pertaining to age [at tetralogy of Fallot repair and at cardiovascular magnetic resonance imaging]) model performance did not change significantly. However, when cardiopulmonary exercise variables were removed, model performance decreased significantly. Strong model performance using readily accessible clinical data suggests that machine learning can be broadly applied for streamlined and enhanced cardiovascular care. Follow-up studies including multiple centers with longer follow-up and serial time points will be required to determine the value of machine learning modelling for risk stratification in repair of tetralogy of Fallot.

Despite successful repair of tetralogy of Fallot (rTOF) in childhood, advancing age is associated with increased morbidity and mortality.^1–4^ Identification of patients at increased risk of adverse clinical outcomes, such as sudden cardiac death or malignant arrhythmia, has been the focus of intensifying research efforts over recent decades. Previously published risk scores for prediction of major adverse cardiovascular events (MACE) have been largely restricted by the number of clinical variables included in the models and have typically incorporated invasive or specialized measures not used in routine clinical practice.^4,5^ As a result, existing models for MACE prediction have been limited by range of clinical applicability and modest predictive capacity.

Artificial intelligence (AI) is a field of computer science, which holds great promise for cardiovascular medicine in terms of enhanced accuracy and improved efficiency for diagnosis of disease and for prediction of adverse outcomes.^6–9^ Machine learning (ML), a subtype of AI, has successfully incorporated an array of unselected variables for prediction of MACE in acquired heart disease.^10,11^ Creation of a risk prediction model with excellent discriminative ability in congenital heart disease, incorporating routinely used and widely available variables, would be highly desirable. In this study, our primary objective was to explore creation of a robust ML model for risk prediction in rTOF using a broad array of clinical and imaging variables applied to a real-world clinical cohort of patients. We hypothesized that the ML model would successfully discriminate 5-year MACE in a relatively unselected group of adults with rTOF.

## Methods

### Data Sharing

The data that support the findings of this study are available from the corresponding author (Rachel.wald@uhn.ca) upon reasonable request.

### Study Population

To first develop and then to validate a ML model, we sequentially used 2 distinct institutional databases of adults with rTOF followed at our center (University Health Network). Our model was initially developed on a prospectively assembled registry of comprehensively phenotyped adults with rTOF and contemporary cardiovascular magnetic resonance imaging (CMR) studies (analyzed by a central reader) who were considered at risk of adverse events due to the presence of at least moderate chronic pulmonary regurgitation (PR); this was designated as the development dataset.^12^ The model was subsequently validated on an independent, retrospective database of adults with rTOF with a full range of PR severities (absent to severe) who had CMR (reflecting multiple readers over a span of decades) using routine variables available from the electronic health record; this was designated the validation dataset. As CMR is considered a cornerstone for stratifying risk and for directing management in the rTOF population, patients without a contemporary CMR were excluded from study analysis. Development and validation datasets were nonoverlapping and as such were comprised of unique individuals (specifically those included for study in the development dataset were excluded from analysis in the validation dataset).

### Data Collection and Image Analysis

**Figure 1.**
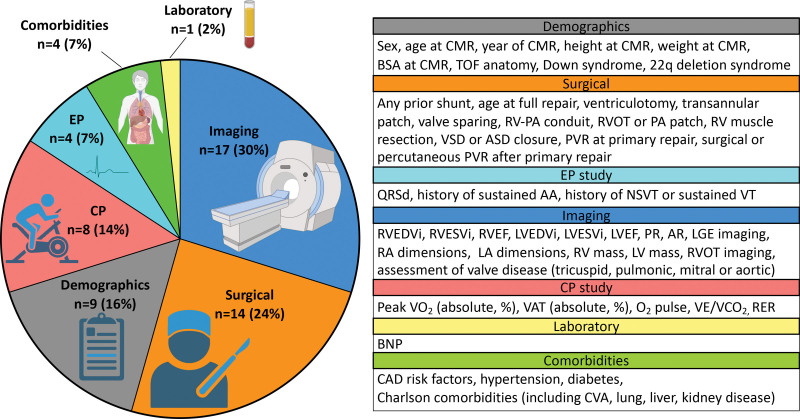
**Clinical and imaging variables incorporated into the machine learning model (n=57 variables).** AA indicates atrial arrhythmia; AR, aortic regurgitation; ASD, atrial septal defect; BNP, brain natriuretic peptide; BSA, body surface area; CAD, coronary artery disease; CMR, cardiovascular magnetic resonance imaging; CP, cardiopulmonary exercise study; CVA, cerebrovascular accident; EDVi, end-diastolic volume indexed; EF, ejection fraction; EP, electrophysiology study; ESVi, end-systolic volume indexed; LA, left atrium; LGE, late gadolinium enhancement; LV, left ventricular; NSVT, nonsustained ventricular tachycardia; PA, pulmonary artery; PR, pulmonary regurgitation; PVR, pulmonary valve replacement; QRSd, QRS duration; RA, right atrium; RER, respiratory exchange ratio; RV, right ventricular; RVOT, right ventricular outflow tract; TOF, tetralogy of Fallot; VAT, ventilatory anaerobic threshold; VE/VCO_2_, minute ventilation/carbon dioxide production relationship; VO_2_, aerobic capacity; VSD, ventricular septal defect; and VT, ventricular tachycardia (portions of this figure were created with Biorender.com)

The spectrum of clinical and imaging variables used for ML are demonstrated (Figure 1). The observation period for each patient was set to begin on the date of CMR (time zero) coincident with study entry for the prospective registry and the earliest completed CMR for the retrospective database. Clinical data (electrocardiograms, echocardiograms, cardiopulmonary exercise studies, and laboratory studies) were identified within 2 years of the index CMR study. Extracardiac disease was classified according to the Charlson Comorbidity Index.^13^ Our methodology for cardiopulmonary exercise study performance and image analysis (CMR and echocardiography) has been previously described in detail.^12^ Briefly, ventricular volumes, function and mass were quantified using CMR. Extent of valvular insufficiency was determined using CMR and severity of valvular stenosis was characterized using echocardiography. Atrial enlargement was preferentially defined as an area using CMR^14^ although echocardiographic quantification of dimensions^15^ was used in the absence of CMR data. Missing data were encoded as −1.0 to direct data available for use in the model which were subsequently handled by AI.

### Major Adverse Cardiovascular Events

The primary composite outcome was comprised of all-cause mortality, resuscitated sudden cardiac death, sustained ventricular tachycardia (>30 seconds), or heart failure (hospital admission >24 hours for intravenous diuretics). Our adjudication process for ascertainment of outcomes has been previously published and was similarly applied to the present study.^12^ Vital status was determined following review of all available records and included telephone calls to confirm date and cause of death, as necessary. To predict 5-year MACE in rTOF, only events within the first 5 years were considered and patients without at least 5 years of follow-up were excluded.^16^ For comparison, a comprehensive model was created using all available patients and their follow-up data.

### Model Development and Validation

**Figure 2.**
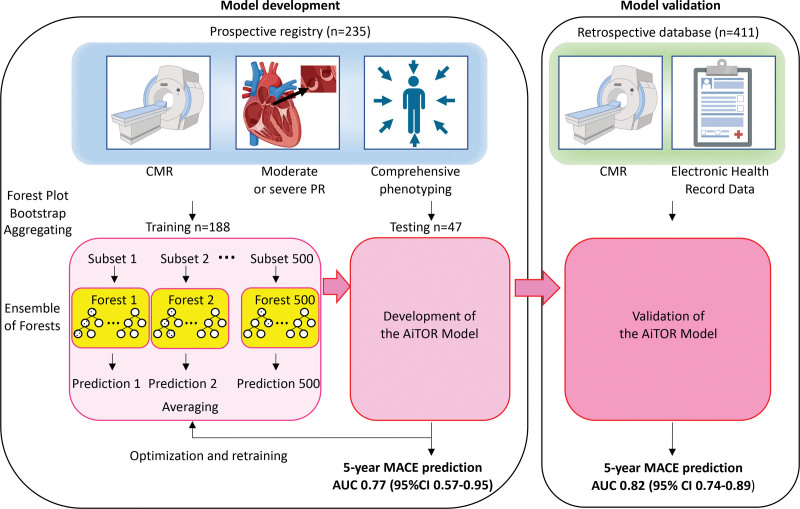
**Random forest plots used to establish the machine learning prediction model for 5-year major adverse cardiovascular events (MACE) prediction.** Sub-datasets of data were initialized using a bootstrap aggregating method for data development. Subsequently, each subset was developed using decision tree analysis by binary recursive splitting of data and average prediction from trees to provide the final MACE prediction model (left). Independent validation without overlapping was accomplished on the validation data (right). AiTOR indicates artificial intelligence model derived from Toronto patient data; AUC, area under the curve; CMR, cardiovascular magnetic resonance imaging; and PR, pulmonary regurgitation (portions of this figure were created with Biorender.com)

The random forest algorithm was the ML technique selected for model development. Random forests are a nonparametric ML model that use an ensemble (the forest) of models (trees) to robustly model structured data. Tree ensemble models have been demonstrated to generally outperform deep learning for tabular data.^17^ The random forest algorithm’s chief strengths are that as a nonparametric method it makes no assumption of how the data are distributed, and as ensemble it leverages bagging to avoid overfitting to outlier features and bootstrapping to avoid overfitting to outlier data samples (patients). Its weakness is that the model is less easy to characterize than multivariate linear regression or a Cox model. Briefly, a total of 500 sub-datasets were initialized using a bootstrap aggregating method with repeated random stratified sub-sampling of the development dataset into disjoint sets with 80% of the dataset for model training and 20% of the dataset for model testing. Each subset in the decision tree was developed following binary recursive splitting of data along with averaging of prediction from forest to provide the final model for MACE prediction. Model validation was subsequently performed on the distinct validation dataset (Figure 2). The predictive capacity of each model for MACE at 5 years was defined using receiver-operating characteristic analysis characterized by the area under the curve (AUC) along with 95% CIs. The Harrell concordance index (C-index) was used to explore time-independent model performance with inclusion of all patients irrespective of follow-up duration. A feature map using Shapley Additive exPlanation (SHAP) values was created to demonstrate the relative contribution of each variable within the final AiTOR model (artificial intelligence model derived from Toronto patient data) with designation of risk according to color (red corresponding to high risk and blue corresponding to low risk) and the length of the horizontal bar indicating feature importance. Briefly, SHAP uses a game theoretical approach to interrogate a model and determine what risk level it would predict with and without access to a given feature. The larger the gap the more important the feature to the model. By further examining a model’s prediction per patient and with or without each particular value of a feature in a training set (eg, if the model knows the patient’s right ventricular [RV] ejection fraction is 19 versus not knowing it at all, how does the risk change?), the SHAP values can further characterize the dependence of risk on a range of feature values.

### Comparison of Model Performance Against Existing Risk Scores

Performance of the AiTOR model was compared against previously established risk scores for prediction of adverse clinical outcomes in rTOF, with restriction of variables to those used in routine clinical practice for disease surveillance in rTOF without inclusion of invasive or specialized parameters or measures. Specifically, scores were assigned as described by Khairy et. al (including prior palliative shunt, previous ventriculotomy, QRS duration ≥180 msec, and nonsustained ventricular tachycardia, but excluding invasive electrophysiological stimulation data and invasive left ventricular end-diastolic pressure measurements),^5^ by Bokma et. al (defined as the noninvasive Khairy score as described above with the addition of CMR-derived right ventricular and left ventricular ejection fraction [RVEF, LVEF])^18^ and by Ghonim et. al (including age >50 years, sustained atrial arrhythmia, brain natriuretic peptide [BNP], peak oxygen uptake on exercise testing, and CMR-derived RVEF and LVEF but excluding late gadolinium enhancement [LGE]); LGE continues to be relatively restricted in clinical practice as a parameter reserved for targeted indications on a per patient basis as opposed to routine surveillance.^4^ Risk scores were calculated exclusively using patients from the validation dataset to broaden generalizability given the real-world nature of this relatively unselected dataset.

### Statistical Analysis

Continuous variables are presented with mean (SD) and median (IQR), as appropriate. Categorical data are reported using frequency and percent. Comparisons between groups were made with the Student t test or Wilcoxon Rank-sum test for continuous variables and the χ^2^ test or Fisher exact test for discrete variables, as appropriate. A multivariable Cox regression model with elastic net penalty was used for conventional risk assessment. Receiver-operating curves generated by different models were presented as AUC with 95% CIs and compared using a 2-sided DeLong method. A *P*<0.05 was considered statistically significant. Analyses were conducted using JMP Pro.16 statistical software package (SAS Institute, North Carolina, USA). Models were developed in Python 3.9 using scikit-learn for random forest algorithms and scikit-survival for Cox modelling. SHAP analysis was performed using the Shap Python package. This study was approved by our institutional research ethics board; informed consent was obtained for prospectively enrolled patients (study number 12-0242) and a waiver of consent was granted for retrospectively studied patients (study number 20-5873).

## Results

### Patient Characteristics

**Figure 3.**
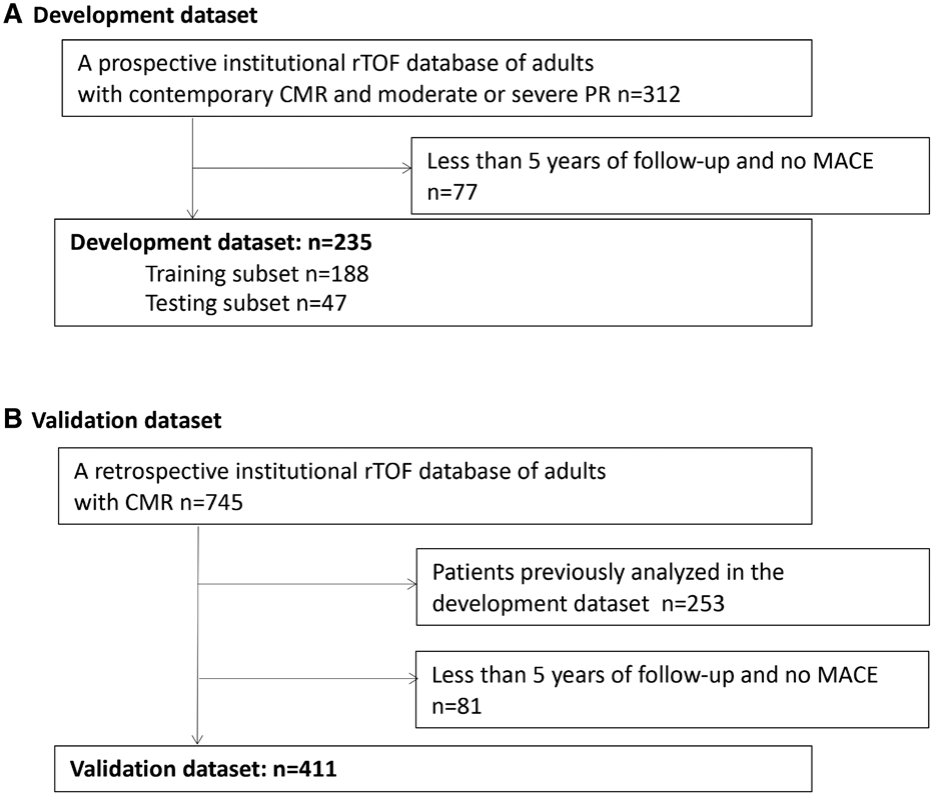
**Cohort diagrams. A**, A prospective institutional database of patients with repaired tetralogy of Fallot (rTOF) was the basis of the development dataset used to predict 5-year major adverse cardiovascular events (MACE) with exclusion of patients without a minimum of 5 years of follow-up and without MACE. **B**, A retrospective institutional rTOF database was the basis of the validation dataset used to predict 5-year MACE. Patients previously analyzed in the development dataset were excluded. Patients without a 5 year minimum follow-up duration and without MACE were excluded. CMR indicates cardiovascular magnetic resonance imaging; and PR, pulmonary regurgitation.

A total of 804 unique patients ≥18 years of age with rTOF and follow-up at our institution in Toronto were identified. There were 312 patients in the development dataset (59% male, mean age 34±13 years, median follow-up duration 6 years [IQR 4-7]) and 492 patients in the validation dataset (58% male, mean age 30±12 years, median follow-up 11 years [IQR 6-17]). For prediction of 5-year MACE, a total of 646 patients were included. Specifically, there were 235 patients in the development dataset (57% male, mean age 34±13 years, median follow-up duration 5 years [IQR 5-5]) and 411 patients (57% male, mean age 30±12 years, median follow-up duration 5 years [IQR 5-5]) in the validation dataset (Figure 3). The number of datapoints available for each variable included in the 5-year model are summarized (Table 1) and baseline patient characteristics are shown (Table S1).

**Table 1.**
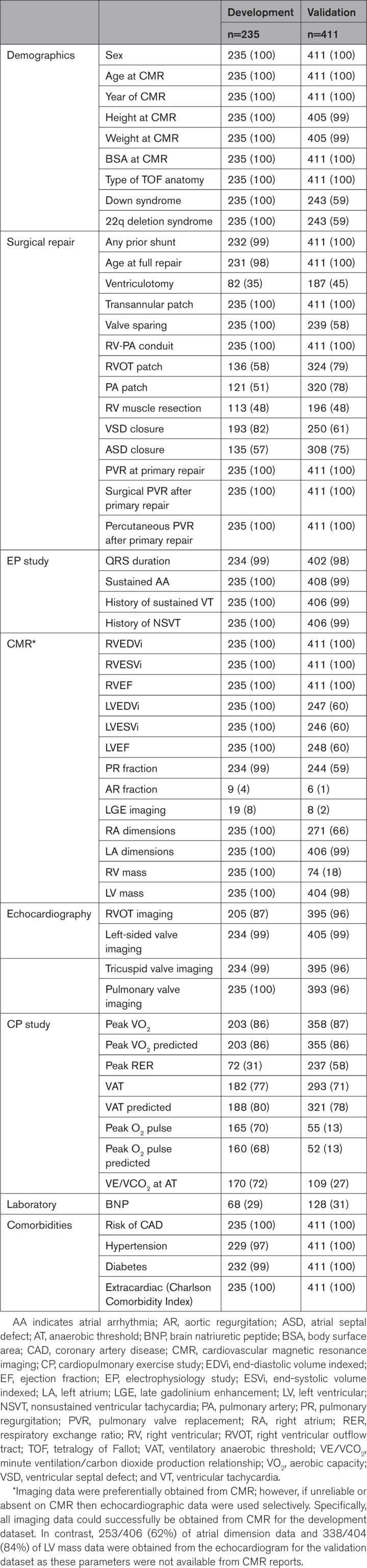
Datapoints Available for Analysis in Development and Validation Datasets for 5-Year Prediction of Major Adverse Cardiovascular Events (Counts With Percent; n=57 Variables)

### Major Adverse Cardiovascular Outcomes

At 5 years of follow-up, the composite MACE end point was observed in 10% (n=24) of patients in the development dataset and 12% (n=49) in the validation dataset; MACE frequencies did not differ statistically between the development and validation datasets (*P*=0.509). Nonmutually exclusive adverse events are shown (Table 2). One patient in the development dataset and 7 in the validation dataset experienced more than 1 event. Of the 8 deaths in the development dataset, 6 (75%) were cardiovascular, 1 was noncardiovascular, and 1 was of uncertain etiology. Of the 23 deaths in the validation dataset, 16 (70%) were cardiovascular, 3 were noncardiovascular, and 4 were of uncertain etiology.

**Table 2.**
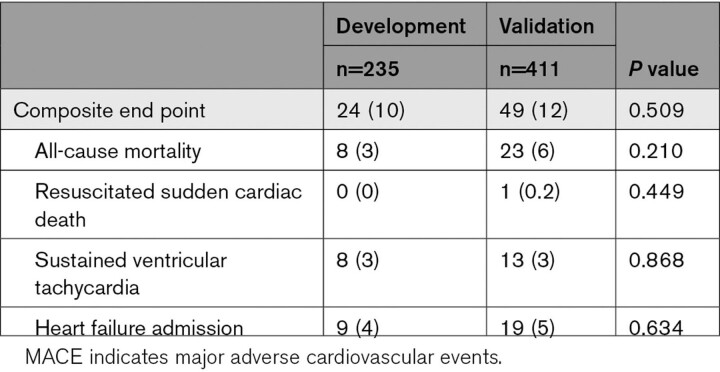
MACE in Development and Validation Datasets (Nonmutually Exclusive Events) for Prediction of 5-Year MACE (Counts With Percent)

### Model Performance

**Figure 4.**
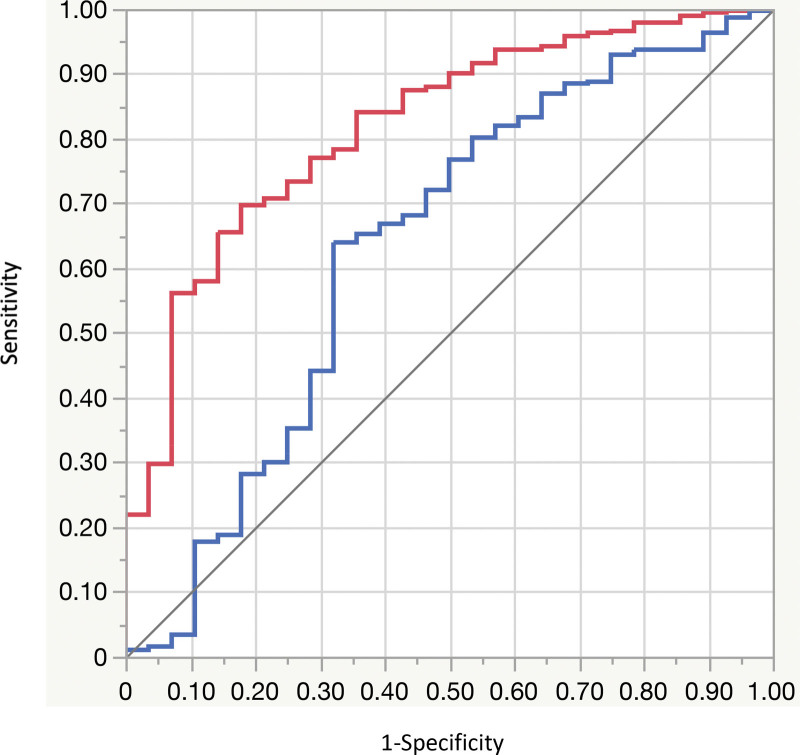
**Comparison of performance between the artificial intelligence model derived from Toronto patient data (AiTOR) and a conventional Cox model.** The AiTOR model is shown in red and the Cox model is shown in blue.

**Figure 5.**
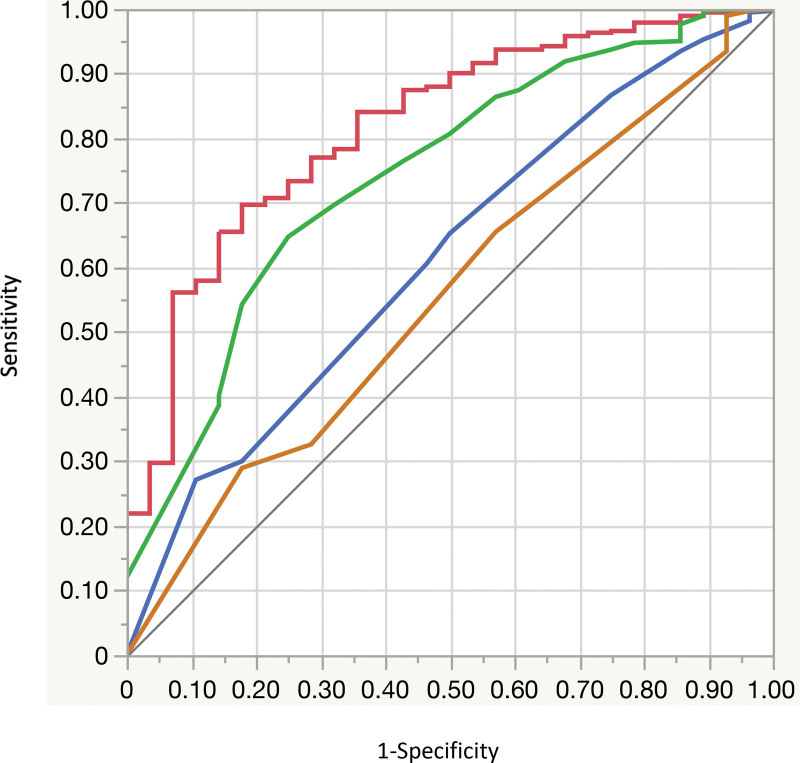
**Comparison of performance between the artificial intelligence model derived from Toronto patient data (AiTOR) model and existing risk scores.** The AiTOR model (shown in red) is compared with previously published risk scores (Khairy score in orange, Bokma score in blue and Ghonim score in green). Components of the risk score are detailed in the text.

The performance of the AiTOR model initially derived from the development dataset for 5-year MACE prediction using prospectively collected data was found to be acceptable (AUC, 0.77 [CI, 0.57–0.95]). Subsequently, the discriminatory capacity of the AiTOR model for MACE at 5 years using routinely available variables from the validation dataset was found to be strong (AUC, 0.82 [CI, 0.74–0.89]) and was superior to a companion model created using conventional multivariable Cox proportional regression analysis (AUC, 0.63 [CI, 0.51–0.75]; *P*=0.003; Figure 4). Comparisons between the AiTOR model and established risk scores are demonstrated (Figure 5). As compared with the Khairy score (AUC, 0.59 [CI, 0.51–0.67]) and the Bokma score (AUC, 0.63 [CI, 0.55–0.71]), the AiTOR model had significantly better predictive performance (*P*<0.001 and *P*=0.001, respectively). Although the AiTOR model performance appeared to have slightly better discrimination than the Ghonim score (AUC, 0.76 [CI, 0.67–0.82]), this difference did not meet statistical significance (*P*=0.096). The C-index for the AiTOR development model (0.79 [CI, 0.63–0.93]) and validation model (0.77 [CI, 0.70–0.84]) confirmed that the model retained good predictive capacity independent of follow-up time.

### Feature Importance

**Figure 6.**
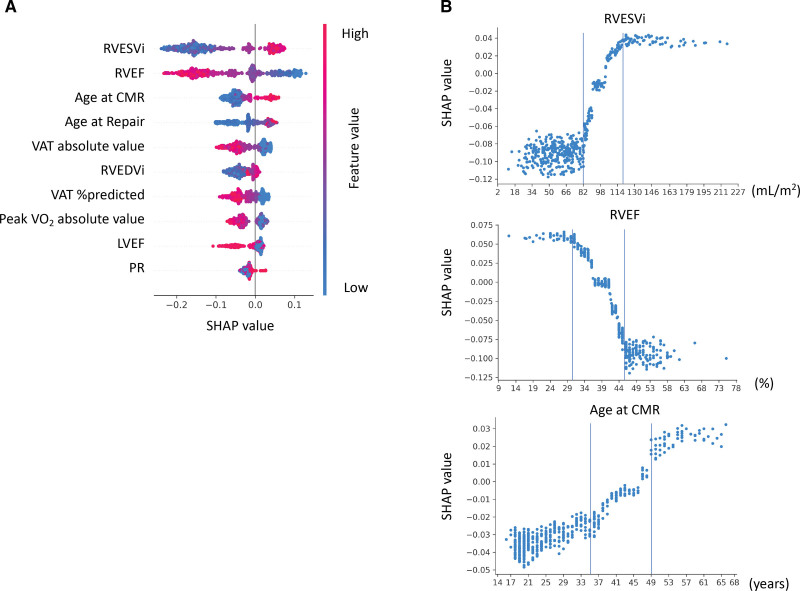
**Feature map demonstrating the strongest variables for prediction of 5-year major adverse cardiovascular events (MACE) and selected plots to demonstrate threshold values where MACE escalates. A**, Demonstration of SHAP values for the 10 strongest features in the artificial intelligence model derived from Toronto patient data (AiTOR) model based on highest mean absolute SHAP values. Each sampling of the test set is represented as a data point per feature, and the *x*-axis shows the positive or negative effect on model prediction based on a specific feature. The color coding depicts the value of the feature and is scaled independently based on the range observed in the data. Designation of risk is according to color (red corresponds to high risk and blue corresponds to low risk) and length of the horizontal bar assigned to respective variables. For example, larger right ventricular end-systolic volume indexed (RVESVi), lower right ventricular ejection fraction (RVEF), and older age at cardiovascular magnetic resonance imaging (CMR) were identified as high-risk features for MACE. Specifically, if for a given patient the model predicts 80% chance of an event with a feature, but 60% chance of an event without having observed that feature, then the SHAP value will be 20% (ie, 0.20). **B**, Plots to depict range of values associated with the greatest change in risk of MACE for selected variables. In the 2 regions of relatively little change (flat zones), MACE risk is not expected to change despite amelioration of a specific parameter; in the region of more pronounced change (slope), MACE risk can be expected to change in accordance with parameter change (ie, increase in RVEF or decrease in RVESVi). LVEF indicates left ventricular ejection fraction; PR, pulmonary regurgitation; RVEDVi, right ventricular end-diastolic volume indexed; VAT, ventilatory anaerobic threshold; and VO_2_, aerobic capacity.

A feature map depicting the relative importance of the variables within the AiTOR model in the validation dataset is shown (Figure 6A). The 10 strongest variables for model prediction were similar in development and validation datasets with slight variations in relative importance (Figure S1). In decreasing order of strength, the variables in the validation dataset were: RV end-systolic volume indexed (ESVi), RVEF, age at CMR, age at TOF repair, ventilatory anaerobic threshold (VAT) absolute, RV end-diastolic volume indexed (EDVi), VAT % predicted, peak aerobic capacity, LVEF and PR fraction. These variables are displayed with their relative impact on the model for event prediction probability, analogous to how confident the model is that a patient will/will not have an event with/without including that feature in the model. Risk increase for the strongest variables, based on model performance, is shown graphically (Figure 6B). Specifically, MACE risk increased most rapidly at the following thresholds: RVESVi 82 to 122 mL/m^2^, RVEF 30 to 45%, and age 35 to 49 years. Restricting the number of variables to only 10 (as compared with the full complement of 57 variables) did not significantly alter AiTOR model performance (AUC, 0.81 [CI, 0.72–0.89] as compared with AUC, 0.82 [CI, 0.74–0.89]; *P*=0.232). However, restricting the model to age and CMR variables only, with elimination of the exercise parameters, yielded inferior model performance (AUC, 0.75 [CI, 0.65–0.84]) as compared with the more complete model (*P*=0.002).

## Discussion

In this study, we describe a novel AI model for prediction of adverse clinical outcomes in rTOF developed and validated using ML techniques. The key findings of this study include the following: (1) the AiTOR model developed from clinical and imaging variables had strong predictive capacity for MACE in rTOF at 5 years which matched or exceeded the discrimination of previously published risk scores when applied to our study population; (2) AiTOR model performance was strong despite the real-world nature of the validation dataset comprised solely of routine variables obtained from our institutional electronic health record without inclusion of invasive or specialized measures; and (3) AiTOR model strength was retained even after restriction to the ten strongest variables (n=5 CMR measures, n=3 exercise parameters and n=2 demographic variables) despite model training and testing using a wide array of clinical and imaging variables (n=57).

### Previously Published Risk Scores

Accurate prediction of sudden death in rTOF has been the focus of intensifying research efforts over recent decades. As compared with the older population of subjects with acquired heart disease, the expanding survivorship of the relatively young rTOF population may shoulder a substantial cumulative impact of risk over the course of a lifetime. The widely recognized and often quoted inception risk score for appropriateness of implantable cardiac defibrillator shock therapy for primary prevention published by Khairy et al in 2008 was based, at least in part, on invasive measures such as stimulation testing for ventricular tachycardia during electrophysiology testing and direct pressure measurements obtained during cardiac catheterization, which may be only occasionally available.^5^ In an attempt to broaden the applicability of the Khairy score and along with the recognition that CMR can provide robust and multi-faceted disease discrimination in rTOF, Bokma and colleagues created a model for prediction of all-cause mortality and ventricular arrhythmia in 2017 using the noninvasive components of the Khairy score along with addition of CMR-derived measures of significant systolic dysfunction (LVEF <45% and RVEF <30%), resulting in a C-statistic of 0.75 (CI, 0.63–0.85).^18^

More recently, Ghonim et al expanded on the predictors of all-cause mortality in rTOF, creating a risk score with inclusion of LGE of the RV and left ventricle in addition to CMR measures of systolic function, exercise parameters, BNP, age, and history of arrhythmia.^4^ Worthy of mention is the weighting of the Ghonim scoring system toward presence of LGE with 40 of the 100 available points assigned in the presence of severe RV LGE rendering this variable the strongest predictor of outcome. Accordingly, the performance of their model was weakened following removal of LGE, decreasing from an AUC of 0.87 (CI, 0.78–0.95) to an AUC of 0.81 (CI, 0.71–0.91). Although indicated for targeted evaluation of specific patients in select circumstances, there are important limitations to intravenous gadolinium-based contrast administration which have prevented widespread adoption of LGE imaging into clinical practice for surveillance in rTOF, some of which include the somewhat invasive nature of contrast injection, the risk of long-term gadolinium deposition in extracardiac structures, contraindication to administration in those with renal insufficiency, and technical difficulties related to performance and interpretation of LGE in the relatively thin-walled and uniquely shaped RV. Although a promising variable in the research arena, LGE in rTOF can be relatively scarce in routine clinical care due to restricted use; in our cohort only 27 subjects (4%) had LGE imaging available for review and as such was the least available of all variables studied (Table 1). Other CMR parameters have been proposed for risk stratification, such as RV mass and RV mass:volume ratio; however, these measures have not been universally incorporated into routine CMR laboratory protocols as of yet.^19^ Although RV mass was available in 100% of the patients studied in the prospective developmental dataset, it was only available in 18% of the retrospective validation dataset and therefore could not be tested by the AiTOR model. Similarly, BNP was missing in a large percentage of patients, available in only 30% (n=196) of our patients attesting to the selective use of this parameter in the clinical (as opposed to the research) setting (Table 1).

### Potential Impact of the Present Study

The design of our newly proposed model and the results of our study have the potential to advance clinical care in several ways. In contrast to many of the previously described models for risk stratification,^4,5,18,19^ our AiTOR model was validated using a noncurated dataset comprised of clinically available measures commonly used in the routine surveillance of adults with rTOF which were extracted from real-world electronic health records of subjects followed in our program. As such, we think that our results may be more widely generalizable than risk scores derived from measures which are invasive in nature and preferentially used in only few select centers.^4,5^ Notwithstanding the broadly available nature of variables used for prediction, the performance of our model was at least as good, and in some instances superior to, the performance of previously established risk scores with an AUC of 0.82 (CI, 0.74–0.89; Figure 5). Additionally, worthy of mention is the alignment between the strongest variables selected by the model and current management guidelines which support routine surveillance CMR and exercise studies with increasing study frequency in accordance with physiological risk profile.

There are several unique strengths related to AI-derived risk scoring, which are noteworthy. Nonlinear models such as those used for ML can identify complex discriminative patterns from large volumes of data without underlying assumptions. Unlike traditional Cox modelling, which identifies cut-points for risk and explores interactions between a selection number of variables in a linear fashion, the ML approach employs more powerful methodology such that ranges of risk can be defined along with dynamic interactions between variables in a nonlinear and nonparametric fashion. Through inherent mitigation properties of the ML algorithm, the nature of the random forest algorithm used in our AiTOR model enabled exploration of a greater number and increased complexity of variables without the typical constraints associated with conventional modelling, such as prior feature selection, limitations to numbers of variables tested and general assumptions of linearity.^20^ Attesting to this fact and in line with previous publications,^10,11^ the random forest plot that applied for ML was superior to traditional linear regression MACE prediction (Figure 4) confirming a more robust approach to risk stratification in the new, as compared to conventional, methods of risk stratification. The top 10 features identified using AiTOR (Figure 6) are entirely in keeping with published risk scores incorporating CMR measures (RVESVi, RVEDVi, RVEF, LVEF, and PR), demographic variables (age), and exercise parameters (VAT, peak aerobic capacity). Specifically, the 3 strongest features, RVESVi,^21,22^ RVEF,^4,18,23^ and age^4^ have been identified as key discriminators of risk in previously published, albeit in disparate, risk scores. Beyond confirmation of existing literature for variable identification, our model extends our understanding of risk prediction by suggesting a range of values where adverse event rates escalate (Figure 6B), specifically at RVESVi 82 to 122 mL/m^2^, at RVEF 30 to 45% and in individuals between 35 and 49 years of age (outside of these ranges, parameter amelioration [such as a decrease in RVESVi or an increase in RVEF] is not expected to translate into modulation of risk). Reassuringly, the AiTOR model maintained strong performance despite restriction to the strongest 10 variables demonstrating an AUC of 0.81 (CI, 0.72–0.89), which was not statistically different from the full model AUC of 0.82 utilizing the full complement of variables (n=57), attesting to the potential portability of the AiTOR model within the clinical setting using a focused list of variables. Weakening of our model following exclusion of exercise parameters underscores the incremental contribution of measures of functional capacity, which are complimentary to imaging measures of ventricular function and size, to risk prediction. Given incorporation of clinical along with imaging variables for prediction of clinically relevant outcomes, our model extends the current body of AI literature in rTOF.^7,24^

### Strength, Weaknesses, and Future Directions

Application of ML techniques can enhance the efficiency and accuracy of adverse event risk prediction in cardiovascular medicine and in this regard our AiTOR model holds promise for possible improvement in rTOF surveillance.^25,26^ In contrast to the previously published risk scores designed to predict all-cause mortality and arrhythmic causes of death,^4,5,18,19^ our risk score is also inclusive of heart failure which enhances its applicability to the contemporary rTOF population who continue to age with associated increasing risk of heart failure. Notably, the validation cohort for our model was constructed from data broadly captured from the electronic health records representing a full spectrum of PR severities with CMR reports from multiple readers spanning decades and as such has potential for broad interpretation and widespread application. However, there are some limitations of this study worthy of mention. While study inclusion required a CMR and as such is in keeping with contemporary guidelines for surveillance in rTOF, high-risk subjects with in-dwelling devices (such as pacemakers or defibrillators) who are known to be at risk for destabilizing arrhythmic events were excluded in keeping with our institutional protocol. While missing data points for some variables (such as BNP and LGE) may impact model discrimination to some degree, we note that this is reflective of the practical nature of our real-world dataset where specialized measures were obtained sparingly as clinically indicated; notwithstanding these limitations, our model has strong predictive capacity, which is enhanced by its promising clinical applicability. Finally, the single-center nature of this study may limit external validity of results, although this is partially mitigated by the fact that the model was developed and validated using 2 distinct and completely independent populations without any decrease in performance from development to validation datasets. Future directions should include validation of this model in a larger cohort from multiple centers, with longer follow-up along with incorporation of serial time points. Ultimately, clinical applicability of this model should be tested with respect to potential for more robust risk stratification with the ultimate goal of improving clinical outcomes in rTOF.

## Conclusions

In this single-center study, a ML-based prediction model comprised of readily available parameters performed well in an independent validation cohort. Further study, ideally incorporating multiple centers with longer follow-up and serial time points, will be required to determine the value of this model for risk stratification and ultimate impact on clinical management in rTOF.

## Article Information

### Acknowledgments

The assistance of Ms Roula Raptis for data management is gratefully acknowledged.

### Sources of Funding

The Canadian Institutes of Health Research (MOP-119353).

### Disclosures

None.

### Supplemental Material

Table S1

Figure S1

## Supplementary Material


