# Supplementary material for: Machine Learning for Prediction of Adverse Cardiovascular Events in Adults With Repaired Tetralogy of Fallot Using Clinical and Cardiovascular Magnetic Resonance Imaging Variables
Source: Circ Cardiovasc Imaging. 2023 Jun 20;16(6):e015205. doi: 10.1161/CIRCIMAGING.122.015205 (PMC10281184; doi:10.1161/CIRCIMAGING.122.015205)
Supplement: Supplementary file 1 [file hci-16-e015205-s001.docx]

**SUPPLEMENTAL MATERIAL.**

**Figure S1.**

**Figure S1. Comparison of the 10 strongest features for model prediction of MACE at 5 years derived from development versus validation datasets.**

The 10 strongest features for prediction of MACE as determined by the development dataset (panel A) and the validation dataset (panel B). CMR, cardiovascular magnetic resonance imaging; LVEF, left ventricular ejection fraction; MACE, major adverse cardiovascular events; PR, pulmonary regurgitation; RVEDVi, right ventricular end-diastolic volume indexed; RVEF, right ventricular ejection fraction; RVESVi, right ventricular end-systolic volume indexed; VAT, ventilatory anaerobic threshold; VO_2_, aerobic capacity.

**Table S1. Clinical variables for prediction of major adverse cardiovascular events using artificial intelligence (counts with percent or mean with SD) (n=57 variables)**

|  |  | Development  n=235** | Validation  n=411** | p value |
| --- | --- | --- | --- | --- |
| Demographics | Sex (male) | 134 (57) | 236 (57) | 0.921 |
|  | Age at CMR (years) | 34±13 | 30±12 | **<0.001** |
|  | Year of CMR (year) | 2014±1 | 2005±5 | **<0.001** |
|  | Height at CMR (cm) | 166±13 | 168±10 | 0.094 |
|  | Weight at CMR (kg) | 73±20 | 72±17 | 0.677 |
|  | BSA at CMR (m^2^) | 1.8±0.3 | 1.8±0.2 | 0.742 |
|  | Type of TOF anatomy (simple) | 173 (74) | 266 (65) | **0.020** |
|  | Down syndrome | 7 (3) | 10 (4) | 0.502 |
|  | 22q deletion syndrome | 16 (7) | 29 (12) | 0.055 |
| Surgical Repair | Any prior shunt | 90 (39) | 204 (50) | **0.008** |
|  | Age at full repair (years) | 5±5 | 7±8 | **<0.001** |
|  | Ventriculotomy | 82 (96) | 173 (93) | 0.211 |
|  | Transannular patch | 137 (58) | 185 (45) | **<0.001** |
|  | Valve sparing | 58 (25) | 84 (35) | **0.016** |
|  | RV-PA conduit | 10 (4) | 48 (12) | **<0.001** |
|  | RVOT patch | 114 (84) | 214 (66) | **<0.001** |
|  | PA patch | 97 (80) | 76 (24) | **<0.001** |
|  | RV muscle resection | 102 (90) | 169 (86) | 0.298 |
|  | VSD closure | 191 (99) | 248 (99) | 0.794 |
|  | ASD closure | 31 (23) | 75 (24) | 0.753 |
|  | PVR at primary repair | 3 (1) | 19 (5) | **0.024** |
|  | Surgical PVR post primary repair | 33 (14) | 249 (61) | **<0.001** |
|  | Percutaneous PVR post primary repair | 4 (2) | 33 (8) | **<0.001** |
| EP study | QRS duration (msec) | 150±26 | 155±26 | **0.012** |
|  | Sustained AA | 32 (14) | 109 (27) | **<0.001** |
|  | History of sustained VT | 6 (3) | 31 (8) | **0.008** |
|  | History of NSVT | 19 (8) | 28 (7) | 0.578 |
| CMR* | RVEDVi (mL/m^2^) | 164±39 | 132±53 | **<0.001** |
|  | RVESVi (mL/m^2^) | 93±28 | 79±36 | **<0.001** |
|  | RVEF (%) | 43±7 | 41±8 | **<0.001** |
|  | LVEDVi (mL/m^2^) | 86±20 | 86±29 | 0.263 |
|  | LVESVi (mL/m^2^) | 40±13 | 42±26 | 0.582 |
|  | LVEF (%) | 54±7 | 54±9 | 0.579 |
|  | PR fraction (%) | 37±14 | 25±20 | **<0.001** |
|  | AR fraction (%) | 3±4 | 6±7 | 0.953 |
|  | LGE imaging | 3 (16) | 1 (13) | 0.826 |
|  | RA dimensions | 33 (14) | 126 (46) | **<0.001** |
|  | LA dimensions | 9 (4) | 71 (17) | **<0.001** |
|  | RV mass | 187 (80) | 47 (64) | **0.005** |
|  | LV mass | 0 (0) | 47 (12) | **<0.001** |
| Echocardiography | RVOT imaging | 67 (33) | 208 (53) | **<0.001** |
|  | Left-sided valve imaging | 31 (13) | 109 (27) | **<0.001** |
|  | TV imaging | 70 (30) | 98 (25) | 0.162 |
|  | PV imaging | 235 (100) | 207 (53) | **<0.001** |
| CP study | Peak VO_2_ (mL/kg/min) | 23.7±9.2 | 24.4±8.7 | 0.272 |
|  | Peak VO_2_ predicted (%) | 69±16 | 64±17 | **<0.001** |
|  | Peak RER | 1.15±0.13 | 1.21±0.14 | **0.002** |
|  | VAT (mL/kg/min) | 14.6±4.9 | 15.1±4.8 | 0.134 |
|  | VAT predicted (%) | 44±12 | 44±12 | 0.546 |
|  | O_2_ pulse (mL/beat) | 10.9±3.2 | 11.1±3.5 | 0.657 |
|  | O_2_ pulse predicted (%) | 85±19 | 77±18 | **0.007** |
|  | VE/VCO_2_ at AT | 35±7 | 34±6 | 0.537 |
| Laboratory | BNP (pg/mL) | 90±157 | 117±155 | **0.006** |
| Comorbidities | Risk of CAD | 64 (27) | 73 (18) | **0.005** |
|  | Hypertension | 16 (7) | 36 (9) | 0.432 |
|  | Diabetes | 5 (2) | 26 (6) | **0.018** |
|  | Extracardiac (Charlson Comorbidity Index) | 33 (14) | 90 (22) | **0.014** |

*Imaging data were preferentially extracted from CMR reports however if unreliable or absent on CMR then echocardiographic data were used selectively

**Details of datapoints available for each variable are shown in Table1

AA, atrial arrhythmia; AR, aortic regurgitation; ASD, atrial septal defect; AT, anaerobic threshold; BNP, brain natriuretic peptide; BSA, body surface area; CAD, coronary artery disease; CMR, cardiovascular magnetic resonance; CP, cardiopulmonary exercise study; EDVi, end-diastolic volume indexed; EF, ejection fraction; EP, electrophysiology study; ESVi, end-systolic volume indexed; LA, left atrium; LGE, late gadolinium enhancement; LV, left ventricular; NSVT, non-sustained ventricular tachycardia; PA, pulmonary artery; PR, pulmonary regurgitation; PV, pulmonary valve; PVR, pulmonary valve replacement; RA, right atrium; RER, respiratory exchange ratio; RV, right ventricular; RVOT, right ventricular outflow tract; SD, standard deviation; TOF, tetralogy of Fallot; TV, tricuspid valve; VAT, ventilatory anaerobic threshold; VE/VCO_2_, minute ventilation/carbon dioxide production relationship; VO_2_, aerobic capacity; VSD, ventricular septal defect; VT, ventricular tachycardia
